# From Biopiracy to Sustainable Knowledge Governance: Epistemic Justice and the Reconstruction of Resource Sovereignty in the Global South

**DOI:** 10.1007/s11948-026-00608-w

**Published:** 2026-06-05

**Authors:** Qi Dong

**Affiliations:** School of History and Culture of Science, Shanghai Jiao Tong University, Shanghai, 200240 China

**Keywords:** Biopiracy, Epistemic justice, Relational sovereignty, Traditional knowledge, Decolonization

## Abstract

Biopiracy represents not merely the exploitation of biological and genetic resources, but a systematic form of epistemic colonialism. This article examines traditional knowledge governance through the lens of epistemic justice and decolonization, integrating Fricker’s theory of epistemic injustice, Kuokkanen’s concept of relational sovereignty, and Santos’s epistemology of the South to construct a three-stage analytical model encompassing “biopiracy, epistemicide, and knowledge sovereignty reconstruction.” Through comparative analysis of institutional innovations in India, Brazil, and South Africa, the study reveals structural exclusion mechanisms inherent in Western property paradigms toward indigenous knowledge while demonstrating fundamental flaws in benefit-sharing mechanisms under the Convention on Biological Diversity framework. The article argues that sustainable knowledge governance must be grounded in epistemic pluralism, achieved through restoring indigenous communities’ epistemic autonomy, recognizing the normative value of relational sovereignty, and establishing polycentric knowledge ecosystems to facilitate a paradigmatic shift from colonial knowledge orders to post-Western knowledge systems.

## Introduction

In an era of the globalized knowledge economy, the illegal appropriation of traditional knowledge, as well as biological and genetic resources has emerged as one of the most contentious issues in North-South relations. Although biopiracy appears superficially as the commercial exploitation of biological resources from developing countries by transnational corporations, it represents, at a deeper level, a more insidious form of systematic epistemic colonialism. This colonialism effects a dual process, from material exploitation to symbolic domination, by transforming the collective intellectual property of indigenous and local communities into private commodities within the Western property rights system. Landmark cases such as the Indian neem tree patent dispute and the Peruvian maca patent controversy reveal a disturbing reality: Western enterprises can circumvent the public domain nature of traditional knowledge and obtain patent protection simply by “scientificizing” traditional uses (Mgbeoji, 2006).

It is important to clarify the analytical boundaries of this inquiry at the outset. As used in this article, “biopiracy” refers specifically to the systematic appropriation of traditional knowledge and biological and genetic resources under conditions of power asymmetry, without adequate free, prior and informed consent and equitable benefit-sharing. This definition does not encompass all forms of cross-cultural knowledge exchange and learning. Knowledge use exists along a spectrum ranging from exploitative appropriation to reciprocal engagement, and where any given interaction falls on that spectrum depends on whether power relations are equitable, whether informed consent is genuinely secured, and whether the agency of knowledge holders is respected. This boundary clarification is essential, as the analysis that follows does not argue against cross-cultural knowledge exchange per se, but rather against the institutional structures that systematically convert such exchange into unilateral appropriation.

Existing research on biopiracy has largely remained within the confines of international legal frameworks and economic benefit distribution, paying limited attention to its underlying nature as a form of epistemic injustice. Santos observes that Western modernity has systematically eliminated the legitimacy of non-Western knowledge systems through epistemicide, relegating traditional knowledge to “primitive experience” or “local knowledge” within the global knowledge hierarchy (Santos, 2014). This epistemic marginalization provides the ideological foundation for biopiracy: when indigenous knowledge is not recognized as “genuine knowledge”, its uncompensated appropriation acquires a concealed legitimacy. The Convention on Biological Diversity (CBD) and its Nagoya Protocol have established benefit-sharing principles, yet their implementation mechanisms, modeled on Western legal templates, often amount to technical repairs rather than fundamental subversions of epistemic colonialism (Robinson, 2010).

The trajectory of multilateral negotiations further illustrates this structural inertia. Since the World Intellectual Property Organization’s (WIPO) Intergovernmental Committee on Intellectual Property and Genetic Resources, Traditional Knowledge and Folklore (IGC) was established in 2000, over two decades of negotiations have failed to produce a binding international instrument for traditional knowledge protection, reflecting the structural reluctance of Northern countries to disrupt existing epistemic power configurations (Oguamanam, 2018). A significant procedural breakthrough occurred in May 2024, when WIPO member states adopted the Treaty on Intellectual Property, Genetic Resources and Associated Traditional Knowledge (GRATK Treaty), establishing a mandatory patent disclosure requirement for genetic resources and associated traditional knowledge (WIPO, 2024). However, this treaty does not confer substantive intellectual property rights upon traditional knowledge holders, nor does it challenge the underlying property paradigm of the existing patent system. As of the time of writing, the treaty has not yet reached the fifteen ratifications required for entry into force. The GRATK Treaty is therefore better understood as an incremental procedural concession within an essentially unreformed epistemic power structure, rather than the paradigmatic shift toward knowledge sovereignty (understood here as the right of indigenous and local communities to autonomously govern their traditional knowledge systems, including collective decisions regarding knowledge access, use, dissemination, and protection) that genuine epistemic justice demands.

This article seeks to transcend conventional benefit-distribution frameworks and re-examine biopiracy and traditional knowledge governance from the perspectives of epistemic justice and decolonization. Three concepts drawn from distinct theoretical traditions are central to the present analysis and require brief clarification for interdisciplinary readers. Epistemic injustice, as formulated by Miranda Fricker (2007), refers to structural mechanisms through which certain knowers are denied credibility on account of social identity prejudice (testimonial injustice) or lack the conceptual resources needed to render their experience intelligible (hermeneutical injustice). Epistemicide, as Boaventura de Sousa Santos (2014) describes it, is “the murder of knowledge”: the systematic destruction of entire knowledge traditions brought about by unequal intercultural exchange. Relational sovereignty, developed by Rauna Kuokkanen (2019) in the context of indigenous self-determination and governance, reconceptualizes sovereignty not as exclusive dominion over things but as the capacity to maintain balance and fulfill responsibilities within relational networks encompassing human communities, non-human beings, and the land. Unlike Western conceptions of sovereignty grounded in territorial exclusivity and atomized individual rights, relational sovereignty is rooted in many indigenous peoples’ understanding that rights and responsibilities are inseparable, and that knowledge, territory, and community relations form an indivisible whole.

By integrating Fricker’s theory of epistemic injustice, Kuokkanen’s concept of relational sovereignty, and Santos’s ecology of knowledges, this study constructs a three-stage analytical model for understanding the evolutionary logic of knowledge governance: the biopiracy stage (resource exploitation), the epistemicide stage (knowledge suppression), and the knowledge sovereignty reconstruction stage (institutional innovation). The research focuses on Global South countries’ institutional practices in defending knowledge sovereignty, exploring alternative governance pathways beyond Western property paradigms through comparative analysis of legislative innovations including India’s Traditional Knowledge Digital Library (TKDL), Brazil’s Biodiversity Law, and South Africa’s Indigenous Knowledge Systems Act. The core argument posits that sustainable knowledge governance must be founded upon fundamental deconstruction of Western epistemic hegemony. Such transformation entails a paradigmatic shift from colonial knowledge orders to polycentric knowledge ecologies through recognition of epistemic plurality and restoration of relational sovereignty.

## Literature Review

Biopiracy emerged as a counter-discourse originally coined by Canadian activist Pat Mooney to challenge developed countries’ accusations of “intellectual property piracy” against developing nations (Mgbeoji, 2006). Shiva (1997) later extended this concept into a broader critique of the colonial logic underlying patent systems, framing the appropriation of indigenous biological and knowledge resources as “the plunder of nature and knowledge”. The concept thus marked a critical shift in thinking about transnational biological resource flows, moving from environmental protection concerns toward the dimensions of knowledge sovereignty and cultural integrity. Early research focused primarily on economic and legal aspects of biopiracy. Aoki (2006) emphasized the complex power dynamics involved, warning against reducing it to technical property rights adjustments. Mgbeoji (2003) further situated biopiracy within a postcolonial critical theoretical framework, revealing its essence as a new form of resource plundering. While these pioneering studies laid foundations for subsequent academic discussion, they focused primarily on material resource flows and economic benefit distribution, with insufficient attention to the power attributes of knowledge itself.

In contrast, another research trajectory demonstrates a clear “epistemic turn.” Dutfield (2004) indicates that the risks of commodifying traditional knowledge extend beyond economic exploitation to threats to cultural integrity and knowledge system autonomy. Through ethnographic research on Latin American bioprospecting projects, Greene (2004) reveals how “cooperation” discourse masks substantive processes of epistemic appropriation, a term denoting not merely the taking of knowledge content, but the forced reconfiguration of knowledge within Western epistemic frameworks, thereby distorting its original epistemological structure and meaning. Ludwig and El-Hani (2025) extend this critique by distinguishing “integrationist” from “transformative” approaches to engaging indigenous knowledge, warning that even well-intentioned transdisciplinary practices can reproduce colonial asymmetries when they treat indigenous knowledge as supplementary data for academic consumption rather than as an autonomous epistemic system with its own frameworks of validation. This line of research has begun to recognize that the roots of biopiracy lie not merely in imperfect legal institutions but in deeper epistemic power inequalities. The marginalized position of indigenous knowledge within modern intellectual property regimes essentially reflects a monopolistic definition of knowledge legitimacy rooted in Western-centrism.

Theoretically, Fricker’s (2007) theory of epistemic injustice provides an important analytical framework for understanding biopiracy. She distinguishes between testimonial injustice and hermeneutical injustice, the former referring to credibility deficits caused by social identity prejudices, the latter to experiences that cannot be understood or expressed due to conceptual resource impoverishment. In traditional knowledge contexts, these two forms of injustice are particularly prominent and mutually reinforcing. Indigenous knowledge holders’ testimonies are systematically undervalued in Western scientific discourse, their knowledge contributions dismissed as “anecdotal evidence” or “folklore” rather than serious epistemic claims. More fundamentally, many indigenous knowledge systems contain concepts that Western scientific paradigms find difficult to accommodate; when such knowledge requires “translation” into Western scientific language for legal protection, its internal logic is often distorted or oversimplified (Agrawal, 2002).

Medina (2013) further develops the concept of “epistemic justice,” emphasizing the democratization of knowledge production and validation mechanisms. He argues that epistemic injustice represents not merely harm to individual cognitive capabilities but a structural power mechanism maintaining epistemic privilege. What knowledge is considered “scientific” and which methods possess “validity” are themselves standards that result from operations of power. The subordinate position of traditional knowledge within modern intellectual property regimes stems not from inferior cognitive value but from evaluation standards established according to Western rationalist and reductionist scientific paradigms. This theoretical insight is crucial for understanding the marginalized status of traditional knowledge under the Agreement on Trade-Related Aspects of Intellectual Property Rights (TRIPS). Article 27 of TRIPS requires patents to meet criteria of “novelty, inventiveness, and utility,” standards that embody the individualistic innovation paradigm institutionalized within the TRIPS framework, though not representative of Western intellectual traditions as a whole, given significant countermovements such as open science, access to knowledge campaigns, and peer-to-peer collaborative models. These criteria fundamentally conflict with the collective nature, gradual evolution, and context-dependency of traditional knowledge (Drahos & Braithwaite, 2002).

Meanwhile, Kuokkanen’s (2019) proposed dimension of “sovereignty’s relational nature” provides a normative foundation for understanding indigenous communities’ rights claims. Unlike conceptions of sovereignty in Western political philosophy based on exclusive territorial control and atomized individual rights, relational sovereignty emphasizes that sovereignty represents not absolute dominion over things but the capacity to maintain balance and fulfill responsibilities within relational networks. This sovereignty conception is rooted in many indigenous cultures’ non-dualistic understandings of human-nature, human-community, and present-ancestor relationships. The North American indigenous “Seven Generations” principle requires current resource decisions to consider impacts on the next seven generations, meaning that contemporary rights to resources are never absolute but constrained by responsibilities to future generations (Lyons, 1980). Applying relational sovereignty to traditional knowledge governance implies profound questioning of existing property paradigms. Traditional knowledge often results from collective creation and intergenerational transmission and cannot be attributed to individual inventors; many indigenous cultures hold that certain knowledge should not be commodified, with inherent dissemination restrictions; and traditional knowledge systems emphasize the relational rather than exchange value of knowledge (Battiste & Henderson, 2000).

Santos’s (2018) concept of “ecology of knowledges” in Southern epistemology integrates epistemic justice and relational sovereignty into a broader decolonizing knowledge politics framework. The core claim of this theory is that different knowledge forms should be understood as an interdependent ecosystem, with no single knowledge form possessing a priori explanatory monopoly over all questions. Western modern science’s global epistemic hegemony stems not from methodological superiority but from historical alliances with capitalist expansion and colonial projects. Colonialism involved not just territorial occupation and resource plundering but the systematic suppression and displacement of non-dominant ways of knowing through educational systems, academic standards, and legal institutions. This epistemic colonialism continues to operate through global education systems, academic evaluation standards, and technical assistance programs even after the end of political colonialism (Quijano, 2007). Santos’s proposed “ecology of knowledges” and “intercultural translation” concepts provide methodological guidance for decolonizing knowledge politics. Mignolo’s (2011) “decolonial turn” theory critically complements this methodological orientation by insisting that decolonization cannot merely be multicultural rhetoric but must address the material foundations of epistemic power. Mignolo’s intervention is analytically significant because it identifies a limitation that Santos’s dialogical methodology does not fully address: the structural constraints that persist even when dialogical methods are pursued in good faith. Epistemic power is not solely a matter of ideas or discursive hierarchies; it is embedded in concrete material and institutional structures, including the dominance of Northern universities in global knowledge production, the gatekeeping role of English-language publishing systems, the evaluative criteria built into international patent examination procedures, and the institutional architecture of bodies such as the WIPO IGC. Without confronting these material foundations, decolonization risks remaining at the level of symbolic recognition, leaving the underlying architecture of epistemic power intact. This argument directly informs the present article’s insistence that sustainable knowledge governance requires structural transformation rather than technical adjustment within existing frameworks, and it establishes the rationale for the empirical focus on institutional practices in the Global South that follows in Sects. “Epistemic Colonial Mechanisms of Biopiracy and Institutional Dilemmas in the Global South” and “Toward Sustainable Knowledge Governance: Normative Principles and Policy Pathways”.

Beyond theoretical critique, scholarship has increasingly examined Global South countries’ agency and institutional innovations in confronting biopiracy. Bavikatte and Robinson (2011) argued early on that institutional innovations often prove ineffective when facing asymmetric global power structures, a diagnosis that has framed much of the subsequent empirical work. Building on this line of inquiry, Eimer’s (2020) comparative research on traditional knowledge protection legislation in India and Brazil indicates that these nations are nonetheless exploring alternative institutional arrangements beyond Western property paradigms. While the literature has made significant progress in theoretical depth and empirical breadth, several clear research gaps remain. First, most research treats epistemic justice, relational sovereignty, and the ecology of knowledges as independent theoretical resources, without systematic theoretical integration. Second, comparative analyses of Global South institutional practices mostly focus on the level of legal texts, with insufficient exploration of practical dilemmas and power constraints. Third, normative discussions about sustainable knowledge governance often remain at the level of general principles, lacking specific institutional design and policy pathway suggestions. This article attempts to address these gaps.

## Theoretical Framework

Constructing a theoretical framework capable of explaining the deep mechanisms of biopiracy while guiding alternative governance practices requires transcending the limitations of any single theoretical perspective. This article integrates Fricker’s theory of epistemic injustice, Kuokkanen’s concept of relational sovereignty, and Santos’s ecology of knowledges into a three-dimensional analytical framework. These three theoretical dimensions are not simply juxtaposed but mutually complementary and progressively deepening in their analytical reach: epistemic justice theory reveals the micro-operational mechanisms of knowledge power, relational sovereignty concepts provide normative foundations beyond Western property paradigms, while the ecology of knowledges offers methodological guidance for constructing pluralistic coexisting knowledge systems.

The integration of these three traditions is necessitated by specific analytical limitations inherent in each. Fricker’s epistemic injustice theory provides precise diagnostic tools for identifying how knowledge marginalization operates, yet it remains fundamentally an analytical theory of justice: it can identify injustice but provides limited resources for articulating what normative foundations an alternative knowledge governance order should rest upon. Santos’s ecology of knowledges offers methodological tools for achieving plural knowledge coexistence, and his work contains important normative dimensions, particularly regarding epistemic justice and knowledge diversity. However, Santos’s normative discussion operates primarily at the macro level of relations between global knowledge systems; it does not sufficiently theorize the specific rights claims at the community level, namely, on what grounds indigenous communities may legitimately govern their knowledge according to their own values and refuse incorporation into external knowledge management systems. Kuokkanen’s relational sovereignty fills precisely this gap by providing an alternative normative foundation rooted in indigenous ontology. Furthermore, relational sovereignty addresses a fundamental epistemological question that neither Fricker nor Santos engages at sufficient depth: the question of the knowledge subject. Western intellectual property frameworks presuppose a specific knowledge ontology in which knowledge is created by identifiable individual subjects, exists independently of context, and can be decomposed into discrete tradable units. This presupposition underwrites the entire logic of the patent system: individual attribution, temporal novelty, universal utility. In many indigenous epistemologies, however, the subject of knowledge is fundamentally different: knowledge belongs not to any individual creator but to a relational network comprising communities, clans, and the broader web of human and non-human relations. Knowledge, land, and community relations are inseparable in indigenous ontology, and governing knowledge is inherently governing this relational totality. When the subject of knowledge is recognized as a relational network rather than an atomized individual, the normative basis of knowledge governance necessarily shifts from exclusive property rights to relational sovereignty. This conceptual migration from Kuokkanen’s original context of indigenous self-determination and territorial governance to the domain of traditional knowledge governance is not an external analogy but an internal extension of the same epistemological foundation. Without this dimension, the analytical framework would have diagnosis (Fricker) and methodology (Santos) but lack the normative bridge between problem identification and institutional reconstruction.

Epistemic injustice manifests in distinctive ways in traditional knowledge contexts. Testimonial injustice most obviously appears in cases of Amazonian indigenous knowledge about plant medicinal properties. Despite millennia of verification and high precision, this knowledge gains recognized value only after laboratory “scientific validation” in modern drug development processes. The pathway from traditional use to pharmaceutical product typically involves isolating active compounds, standardizing extraction procedures, and conducting clinical trials; throughout this conversion process, original knowledge contributions are systematically erased from patent filings and scientific publications, rendering the intellectual labor of indigenous knowledge holders invisible (Posey & Dutfield, 1996). This represents injustice not merely toward individual knowledge contributors but degradation of entire knowledge traditions. Hermeneutical injustice touches deeper issues. Australian Aboriginal “Dreamtime” knowledge systems integrate ecological, social, and spiritual dimensions, rejecting Western science’s subject-object dichotomy and linear causal thinking (Verran, 2002). When such knowledge requires “translation” into Western scientific language for legal protection, its internal logic inevitably becomes distorted. This translation process itself constitutes a new form of epistemic appropriation. A distinct but related pattern of hermeneutical injustice operates at the institutional level in the case of North American indigenous Traditional Ecological Knowledge (TEK). As Whyte (2013) observes, Western environmental governance agencies tend to treat TEK as extractable informational fragments that can be inserted into existing scientific-policy structures, yet TEK functions as a system of relational responsibilities embedded in ongoing community practices. The institutional failure here lies not in cosmological incomprehension but in epistemological assumptions that cannot accommodate knowledge whose validity depends on relational practice. These cases, together with the Amazonian example above, illustrate three analytically distinct dimensions of epistemic injustice: testimonial erasure, cosmological-hermeneutical injustice, and practical-institutional hermeneutical injustice.

As discussed in Sect. “Literature Review”, the TRIPS framework systematically excludes traditional knowledge through its requirements of individual invention, temporal novelty, and universal utility (Drahos & Braithwaite, 2002). The paradox of India’s TKDL further reveals hidden forms of epistemic injustice. To prevent inappropriate patenting of traditional medical knowledge, the Indian government established a digital database containing over 500,000 formulations and practices from Ayurveda, Unani, Siddha, and Yoga, translated into five international languages and classified in international patent classification format, providing major patent offices with prior art references (Ansari, 2020; CSIR, n.d.). This initiative successfully blocked numerous inappropriate patent applications and has been hailed as an innovative model for traditional knowledge protection. However, the TKDL paradox illuminates a deeper form of epistemic injustice: traditional medical knowledge is forced to adopt Western patent system classification logic and scientific language for encoding. An Ayurvedic formulation that in its traditional context encompasses diagnostic considerations of patient constitution, prescriptive guidance tied to seasonal and ritual conditions, and a holistic therapeutic philosophy connected to spiritual practice is, through the TKDL encoding process, stripped of these dimensions and reduced to chemical composition and pharmacological parameters searchable by patent examiners. This process exemplifies what Visvanathan, in his broader critique of cognitive injustice, describes as the “false translation” and “museumization” of living knowledge systems—a form of intellectual apartheid in which traditional knowledge is preserved only by being converted into the discursive form of the regime that devalues it (Visvanathan, 2009). While this “defensive protection” prevents direct piracy, it comes at the cost of accepting Western epistemic hegemony, constituting a new round of erosion of indigenous knowledge’s epistemic autonomy.

The concept of relational sovereignty provides a normative pathway beyond this dilemma. Western property paradigms are built on exclusive territorial control and atomized individual rights; this “absolute sovereignty” paradigm originates from the Westphalian system and Lockean property rights theory, presupposing human-nature dualism and individuals’ absolute dominion over things (Macpherson, 1962). This sovereignty conception, however, is fundamentally at odds with the collective rights concepts and ecological cosmologies of indigenous communities. Many indigenous cultures understand humans as components rather than masters of natural networks, emphasizing stewardship responsibilities rather than ownership. Knowledge in these cultures is not a commodity to be owned and traded but a relational entity that sustains community identity, ecological practices, and cultural transmission.

In contrast to this paradigm of absolute dominion, relational sovereignty emphasizes the capacity to maintain balance and fulfill responsibilities within relational networks. Many Pacific Island nations’ traditional knowledge management systems exemplify this relational logic in practice: certain knowledge can only be transmitted in specific ritual contexts, certain knowledge is limited to specific gender or age groups, and certain sacred site knowledge must not be disclosed to outsiders. These rules function not as “backward restrictions” on knowledge circulation but as cultural mechanisms maintaining knowledge sacredness and community order (Ratuva, 2007). Applying relational sovereignty concepts to biological and genetic resources and traditional knowledge governance implies profound questioning of existing property paradigms. The basic logic of Western intellectual property systems is to incentivize innovation through establishing rights and exclusivity, but this logic encounters fundamental dilemmas in traditional knowledge contexts: traditional knowledge cannot be attributed to individual inventors, certain knowledge should not be commodified or disseminated externally, and propertization may destroy knowledge’s relational value (Anderson, 2009).

Traditional knowledge systems thus present a spectrum of sharing conditions: certain sacred or ceremonially restricted knowledge may be legitimately withheld from external access under communities’ customary governance, an exercise of self-determination rather than a blanket rejection of knowledge exchange. For other forms of traditional knowledge, the issue is not whether to share, but on what terms: whether sharing occurs with free, prior and informed consent; whether the knowledge’s cultural context and relational meaning are respected; and whether benefits flow back equitably to knowledge-holding communities.

The alternative approach of relational sovereignty does not reject all forms of institutional protection but advocates establishing protection mechanisms consistent with indigenous knowledge views. Peru’s Law No. 27,811 on the Protection of Indigenous Peoples’ Collective Knowledge recognizes indigenous communities’ collective rights to traditional knowledge as “perpetual, imprescriptible, and inalienable,” establishing a collective knowledge registration system independent of the patent system (Hepburn, 2020). This legislative innovation reflects an initial recognition of relational sovereignty, though the law still faces conflicts with state sovereignty and private property rights in implementation. When foreign enterprises’ patent rights conflict with indigenous communities’ collective knowledge rights, international trade rules often prioritize protection of the former, while the protective scope of Peruvian domestic law does not extend abroad. This reveals the systematic obstacles that individual Southern countries face in advancing institutional innovations grounded in relational sovereignty under asymmetric global power structures.

Santos’s ecology of knowledges situates these insights within a broader decolonizing knowledge politics framework. Where Fricker diagnoses epistemic harm and Kuokkanen provides an alternative normative foundation, Santos offers the methodological tools for constructing pluralistic knowledge systems. His concept of the “ecology of knowledges” holds that no single knowledge tradition possesses a priori explanatory monopoly; epistemic hegemony is a product of historical alliances between Western science, capitalist expansion, and colonial power rather than of inherent methodological superiority (Santos, 2007).

Decolonizing knowledge politics requires breaking epistemic monism and restoring knowledge diversity. Writing from the standpoint of “subaltern cosmopolitan reason,” Santos proposes three methodologies to challenge the monopoly of Western “lazy reason”: “sociology of absences” reveals mechanisms obscuring marginalized knowledge and practices, activating wasted experiences; “sociology of emergences” focuses on marginal alternative experiments as seeds of systemic transformation; “intercultural translation” builds pluralistic knowledge dialogue based on isomorphic concerns. In practical terms, he critiques anti-globalization movements’ cognitive limitations, advocating the combination of local practices with global solidarity, rejecting savior attitudes, and promoting “alternative globalization” by incorporating marginalized knowledge into education, transforming marginal practices into institutions, and supporting non-capitalist economies, thereby providing pathways for achieving epistemic and social justice (Santos, 2015). Many indigenous peoples’ historical experiences can serve as valuable resources for promoting knowledge diversity. In biodiversity conservation, for instance, the Mi’kmaq concept of Two-Eyed Seeing advocates drawing on indigenous knowledge and Western science simultaneously to understand ecological problems, allowing the two knowledge forms to complement rather than replace each other (Bartlett et al., 2012). Canadian programs applying this method to Arctic ecological monitoring show that this integrated approach yields complementary insights beyond what either scientific data or traditional knowledge alone can provide, enabling more comprehensive understanding of climate change impacts (Gearheard et al., 2010).

However, realizing the ecology of knowledges at institutional levels faces enormous challenges. As Mignolo (2009) argues, so long as the institutional arrangements of global academic production remain dominated by Northern academic institutions, Southern knowledge traditions cannot achieve genuine equal status. Smith (2012) forcefully critiques the continuation of “research as imperialism” in her influential work, advocating research paradigms led by indigenous communities. Some Global South countries have attempted legislative recognition of traditional knowledge’s legal status. Bolivia’s Law of the Rights of Mother Earth, for instance, recognizes nature as a legal subject and grants indigenous knowledge constitutional status; Ecuador’s constitution adopts “Sumak Kawsay” as a development paradigm in place of Western GDP growth models (Villavicencio Calzadilla & Kotzé, 2018). These radical experiments embody a fundamental rejection of Western developmentalism and epistemic colonialism, though they often prove unsustainable when facing international trade rules, transnational capital pressure, and resistance from domestic elite classes.

Synthesizing these three theoretical dimensions, and drawing on the complementary relations elaborated above, this article proposes a three-stage analytical model for decolonizing knowledge governance. The first stage (biopiracy) corresponds to resource plundering and direct epistemic appropriation, typically manifested as transnational corporations commodifying traditional knowledge through patent systems; epistemic injustice theory provides the primary diagnostic tools at this stage, revealing how testimonial and hermeneutical injustice enable knowledge appropriation. The second stage (epistemicide) corresponds to deeper knowledge system suppression, manifested through education systems, academic standards, and legal institutions imposing Western epistemic paradigms as universal standards, causing traditional knowledge to lose its legitimacy foundations; Santos’s critique of epistemicide and the ecology of knowledges provide the structural analysis at this stage. The third stage (knowledge sovereignty reconstruction) represents Global South resistance and institutional innovation, seeking alternative governance pathways through recognizing epistemic justice, practicing relational sovereignty, and establishing knowledge ecology; relational sovereignty provides the normative core at this stage, grounding the reconceptualization of knowledge subjects from individual owners to relational networks. This model describes not only historical processes but also normative exploration of current political possibilities.

This three-stage model is applied in the following sections through a comparative analysis of institutional innovations in India, Brazil, and South Africa, selected for their geographic diversity, distinct legal traditions (common law, civil law, and post-apartheid mixed systems), and divergent approaches to knowledge governance (Eimer, 2020; Wynberg et al., 2009).

## Epistemic Colonial Mechanisms of Biopiracy and Institutional Dilemmas in the Global South

Understanding biopiracy’s epistemic colonial essence requires close analysis of how modern patent systems achieve systematic appropriation of traditional knowledge through seemingly neutral legal techniques. The Indian neem tree case stands as one of the most emblematic disputes in biopiracy history. The neem tree has been used in Indian Ayurvedic medicine for over two millennia, with its leaves, bark, and seeds employed to treat various diseases and as a natural pesticide, and with related knowledge systematically recorded in Sanskrit medical texts (Shiva, 2001). Between 1985 and 1995, American and Japanese enterprises filed over 40 patents related to neem tree extracts, the most controversial being a European patent obtained by the U.S. Department of Agriculture and W.R. Grace Company claiming invention of a “method of controlling fungi on plants using a neem oil formulation.” India strongly protested, noting that these uses had centuries of history in Indian agricultural practices and that patent applicants had merely restated traditional methods in modern chemical language.

After a decade-long legal battle, the European Patent Office finally revoked the patent in 2005, determining it failed to meet “novelty” requirements. However, this victory masks deeper epistemic injustice. Throughout the litigation process, India had to establish traditional knowledge’s “prior art” status through extensive documentary evidence, including records of centuries-old agricultural practices by Indian farmers and prior scientific research on neem that predated the patent application. The burden of assembling and translating such evidence into forms legible to Western patent authorities fell entirely on the challenging parties (Robinson, 2010). The decade-long duration of this challenge and the substantial legal and financial resources it consumed illustrate a structural inequity in the international patent system: the costs of contesting improperly granted patents fall entirely on the communities whose knowledge has been misappropriated, while the original patent applicants bear only relatively modest filing costs. This reveals the first layer of epistemic colonialism in patent systems: knowledge decontextualization. Traditional knowledge is always embedded in specific cultural contexts, ecological practices, and social relations; it understands natural phenomena holistically. Patent systems, however, require decomposing knowledge into patentable “technical information,” a process that systematically erases knowledge’s cultural significance and ecological connections.

The second layer of epistemic colonialism manifests in the individualized attribution of innovation. The Peruvian maca case further highlights this issue. Maca is a traditional crop cultivated by Andean highland indigenous peoples for millennia, possessing nutritional and medicinal value. In the early 2000s, American company Pure World filed multiple patents for maca extracts, claiming invention of “maca extracts and preparation methods for enhancing sexual function” (Landon, 2007). The Peruvian government and indigenous organizations protested that maca’s effects were common knowledge in Andean communities, with the company merely standardizing dried maca powder using modern pharmaceutical processes. However, under the TRIPS framework, traditional cultivation and usage knowledge is not considered “invention”; only standardized extraction processes meet patent requirements. As Dutfield (2001) has detailed, the TRIPS system’s criteria for patentability were designed with industrial innovation in mind and contain structural biases against knowledge produced through collective, incremental, and context-dependent processes. This outcome hinges on the patent law requirement of “non-obviousness” (US patent law) or “inventive step” (European patent law), which demands that an invention not be obvious to a person skilled in the art given the existing prior art. In principle, this criterion should prevent the patenting of well-known traditional practices. In practice, however, because traditional knowledge is often documented in local languages, transmitted orally, or embedded in cultural practices not indexed in patent databases, it remains invisible to patent examiners as prior art. The same knowledge, once repackaged through modern extraction or standardization processes, thereby paradoxically satisfies the very non-obviousness requirement that should have precluded patenting in the first place. This institutional design permanently relegates traditional knowledge holders to “raw material supplier” status: they provide millennia of breeding knowledge and usage experience yet are excluded as innovation contributors in the value chain.

The third layer of epistemic colonialism is forced value commodification. Patent systems presuppose that the purpose of knowledge is commercial development and market exchange, while traditional knowledge’s value orientations often differ radically. For Andean farmers, maca represents not just nutritious food but a symbol of cultural identity, an element of community rituals, and an outcome of ecological adaptation. Reducing it to a “sexual function enhancing commodity” constitutes crude degradation of its cultural significance (Landon, 2007). The deeper issue is that patent systems forcibly require knowledge to enter market exchange logic for legal protection, creating a dilemma for indigenous communities that refuse to commodify certain sacred knowledge. For these communities, the refusal to commodify is not a conservative restriction on knowledge circulation, but an act of preserving the ontological character of knowledge as relational existence; its normative basis lies in the self-determination of communities as maintainers of relational networks.

The epistemic colonial mechanisms embedded in patent systems do not operate in isolation. Market forces amplify and extend the dispossession that formal institutional structures initiate, sometimes producing effects that patent reform alone cannot remedy. The case of quinoa illustrates how the commercialization of traditional biological resources, even without patent monopolies, can deprive knowledge-holding communities of access to their own resources. After quinoa was promoted as a “superfood” in Northern markets beginning around 2006, surging international demand caused prices to rise sharply, rendering this ancestral crop unaffordable for many consumers in Peru and Bolivia who had relied on it for generations. This case demonstrates that exploitative appropriation of traditional knowledge and biological resources operates not only through the legal architecture of patent systems but also through the price mechanisms of global markets, constituting a form of structural dispossession beyond the reach of intellectual property reform alone.

The institutional dimension of this exploitation involves a fundamental asymmetry in the distribution of protection costs. Under the current international patent system, applicants, typically transnational corporations with substantial legal and financial resources, bear relatively modest filing fees to secure exclusive rights. When those “inventions” are grounded in traditional knowledge, however, the burden of proof and the costs of litigation to challenge improper patents fall on knowledge holders with far fewer economic and legal resources. India invested a decade and significant public expenditure to overturn a single neem patent. The TKDL comprises over 500,000 formulations and practices translated into five languages and classified according to international patent standards (Ansari, 2020; CSIR, n.d.). Establishing and maintaining this database represents an enormous human and financial investment borne entirely by the Indian government rather than shared by the patent applicants who benefit from traditional knowledge. In the basmati rice case, India could only exert pressure through diplomatic channels and international public opinion, lacking effective legal remedies (Robinson, 2010). This amounts to a system in which those whose knowledge is misappropriated must pay for their own defense, a structural feature that constitutes the economic dimension of epistemic injustice.

Facing such systematic epistemic colonialism, Global South countries have not remained passive but have actively pursued institutional innovations. India’s traditional knowledge protection system encompasses both defensive and positive protection dimensions. Beyond the TKDL, India’s Biodiversity Act establishes a three-tier management system: National Biodiversity Authority, State Biodiversity Boards, and local Biodiversity Management Committees (BMCs). The Act empowers local communities to establish BMCs for documenting and managing local biodiversity and associated traditional knowledge, though in practice BMCs are granted only consultation rights rather than genuine free, prior, and informed consent authority over access decisions (Joshi et al., 2025). This design nominally gestures toward relational sovereignty ideals, yet falls short of recognizing knowledge rights as inherent to communities. By 2025, India had established over 276,000 BMCs across states and union territories, constituting the world’s largest-scale community knowledge governance experiment in formal terms (NBA, n.d.). However, BMCs face multiple structural challenges: they are typically constituted at the Panchayat/Municipality level rather than at the level of individual indigenous settlements, and thus often do not represent the actual rights and knowledge holders; many communities also lack the technical and legal resources for effective negotiation with commercial actors (Joshi et al., 2025).

Brazil has adopted a different institutional path, emphasizing greater state sovereign control over biological genetic resources. Brazil’s Biodiversity Law continues the CBD’s “resource state sovereignty” principle, defining biological and genetic resources as state assets requiring registration with the Genetic Heritage Management Council for any access (da Silva & Oliveira, 2018). Unlike the Indian model, Brazil’s prior consent rights are primarily exercised by the state rather than communities, requiring prior consent from relevant indigenous or local communities only when involving “identifiable source” traditional knowledge. This design reflects Brazil’s emphasis on state sovereignty but has also drawn criticism from indigenous organizations for weakening community autonomy. Brazil’s innovation lies in its flexible benefit-sharing mechanisms, with benefits taking forms including monetary compensation, technology transfer, capacity building, and community development projects. More importantly, the law establishes a national benefit-sharing fund where benefits enter the fund for overall traditional knowledge protection and indigenous community development when traditional knowledge sources cannot be confirmed to specific communities (Folgosi et al., 2021).

South Africa’s Protection, Promotion, Development and Management of Indigenous Knowledge Act 6 of 2019 represents a more radical decolonization attempt. The Act explicitly places traditional knowledge protection within a broader historical justice framework, reflecting the 2004 Indigenous Knowledge Systems Policy’s position that colonialism and apartheid systematically devalued, suppressed, and deprived African indigenous knowledge systems, and that restoring these knowledge systems’ dignity and value constitutes part of the democratic transition (Department of Science and Technology, South Africa, 2004). This formulation politicizes traditional knowledge issues as reckoning with colonial legacies rather than technical property adjustments. South Africa’s framework adopts a more holistic approach than India and Brazil, not only protecting indigenous knowledge from unauthorized use, misappropriation, and misuse, but actively promoting indigenous knowledge’s wider application in new products, services, and processes. South Africa’s institutional innovations include establishing the National Indigenous Knowledge Systems Office (NIKSO) to register, catalogue, and document indigenous knowledge; recognizing indigenous knowledge as prior art under intellectual property laws; regulating equitable benefit distribution; and certifying indigenous knowledge practitioners (Zondi, 2021).

Comparing these three countries’ institutional innovations reveals common characteristics and systematic obstacles in Global South knowledge sovereignty reconstruction. Common characteristics include transcending Western individualistic property models and recognizing the legitimacy of collective rights; emphasizing prior informed consent principles as a procedural embodiment of relational sovereignty; exploring non-commodified benefit-sharing methods; and establishing multi-level governance structures. However, they all face systematic obstacles from asymmetric global power structures. Most fundamental is the “strong TRIPS, weak CBD” configuration. The TRIPS Agreement, as part of the WTO’s dispute settlement mechanism, is mandatory, with TRIPS violations potentially facing trade sanctions; conversely, while the CBD and its Nagoya Protocol establish benefit-sharing principles, they lack effective enforcement mechanisms (Greiber et al., 2012). The basmati rice case discussed above further illustrates how this configuration operates in practice: even when a Southern country successfully challenges a patent, the absence of a binding multilateral enforcement mechanism means that protection depends entirely on case-by-case contestation within Northern patent systems.

The second obstacle is regulatory absence in Northern consumer markets. Even if Southern countries establish strict access and benefit-sharing systems, regulatory absence in Northern markets allows illegally accessed products to circulate freely. While the Nagoya Protocol requires parties to establish “user compliance” regulatory systems, enforcement of these provisions depends heavily on parties’ political will (Morgera et al., 2014). The third obstacle is social differentiation and elite capture within Southern countries. Traditional knowledge protection legislation is often dominated by urban elites whose understanding of rural communities and indigenous knowledge systems tends to be limited. In India, BMC establishment in some regions has become a new instrument for local elites to consolidate control over resources, with genuine knowledge holders excluded from decision-making (Joshi et al., 2025). The fourth obstacle concerns new challenges in the digital age. With dramatically declining gene sequencing costs and synthetic biology’s rise, biopiracy is shifting from physical resources to Digital Sequence Information (DSI). Southern countries’ access and benefit-sharing laws primarily target physical resources, leaving vast gray areas in DSI jurisdiction (Nehring, 2022).

It is also important to acknowledge the historical complexity of global knowledge flows. Cross-cultural knowledge exchange, including the kinds of crop dissemination exemplified by the spread of sweet potatoes from Peru to South Asia or plantains from Southeast Asia to the Caribbean, has existed throughout human history and has brought profound benefits to regions across the Global South. The analysis in this article does not deny this historical reality. Rather, it focuses on a specific contemporary problem: how, following the establishment of modern intellectual property regimes, particularly the TRIPS system, knowledge flows under conditions of power asymmetry have been transformed from reciprocal exchanges into institutionalized unilateral appropriation. Santos’s critique of epistemic colonialism illuminates a further, often overlooked dimension of this asymmetry: colonial educational legacies continue to shape knowledge production patterns within the Global South itself, manifesting in a persistent reluctance among Southern scholars to collaborate directly with one another without Northern intermediaries, and in a broader lack of awareness of how much nations at similar latitudes have historically benefited from mutual knowledge exchange. Addressing these internal dimensions of epistemic colonialism constitutes an important and still underexplored frontier of decolonizing knowledge politics.

## Toward Sustainable Knowledge Governance: Normative Principles and Policy Pathways

Based on the preceding theoretical analysis and empirical examination, this section proposes a normative framework for sustainable knowledge governance and explores policy implementation pathways at different levels.

The “sustainability” of sustainable knowledge governance carries triple meanings: ecological sustainability (protecting biodiversity and cultural diversity upon which knowledge depends), social sustainability (ensuring intergenerational and community equity in knowledge benefits), and epistemic sustainability (maintaining the diversity and autonomous development capacity of knowledge systems). Before elaborating specific governance principles, it is worth noting that sustainable knowledge governance does not aim to seal off knowledge flows or reject all forms of cross-cultural knowledge exchange. On the contrary, several fields have demonstrated that respectful engagement with traditional knowledge can actively revitalize knowledge systems. Various schools of agroecology, for instance, have done commendable work in recognizing smallholder inventiveness, giving proper credit to local practitioners, and systematically examining which farming practices prove effective and transferable to other regions, particularly among communities at similar latitudes in the Global South. Likewise, participatory plant breeding programs (Ceccarelli & Grando, 2007), in which farmers and scientists collaborate as co-creators of crop varieties, have shown that local agronomic knowledge and formal scientific methods can interact on equitable terms without the former being subsumed into the latter. The institutional conditions advocated below, including free, prior and informed consent, community self-determination, and non-commodified benefit-sharing, are precisely the mechanisms that distinguish such respectful engagement from exploitative appropriation, and that determine where any given knowledge exchange falls on the spectrum between these two poles.

The primary principle of sustainable knowledge governance is epistemic equality. This does not mean all knowledge claims are equally correct or valid, but that no knowledge tradition naturally possesses explanatory monopoly over all questions. At the policy level, this requires establishing pluralistic knowledge validation mechanisms. Current patent examination, research review, and policymaking rely almost exclusively on Western scientific standards, necessitating parallel mechanisms inclusive of indigenous knowledge validation methods. The validity of certain traditional ecological knowledge depends on long-term observation and holistic understanding and cannot be verified through short-term laboratory testing; such knowledge requires alternative validation standards based on intergenerational transmission records and community consensus (Jessen et al., 2022). Although the institutional conditions differ significantly from those in the Global South, certain policy experiments in settler-colonial states offer instructive, if partial, models for operationalizing such epistemic plurality. Canada’s Northwest Territories’ Traditional Knowledge Policy, adopted in 1997, recognizes indigenous traditional knowledge as a valid and essential source of information about the natural environment and requires its incorporation into government decisions on land use and resource management (Keats & Evans, 2020). In New Zealand, the Te Awa Tupua (Whanganui River Claims Settlement) Act 2017 granted legal personhood to the Whanganui River based on Maori relational ontology and established a co-governance body with one Crown-appointed and one Indigenous community-appointed representative, embedding indigenous cosmological principles directly into statutory environmental governance (Charpleix, 2018). These cases illustrate that institutional mechanisms for epistemic equality are not merely theoretical aspirations but have been translated into operational governance frameworks, though their transferability to Global South contexts requires careful attention to differences in state capacity, legal tradition, and the political standing of indigenous communities.

The second principle is epistemic autonomy, which derives directly from the relational sovereignty framework elaborated in Sect.  3. If the subject of knowledge is a relational network rather than an individual, then the right to govern knowledge naturally belongs to the communities that sustain that relational network, not to external legal systems. Epistemic autonomy thus represents the epistemic dimension of relational sovereignty: indigenous and local communities have the right to manage their knowledge according to their own values and norms rather than being forcibly incorporated into external knowledge management systems. This principle encompasses both the right to share and the right not to share; both dimensions are equally important, and both rest on the premise of community self-determination rather than external imposition. Although no existing legal framework fully realizes this principle as an inherent right, certain mechanisms have begun to approximate its institutional logic. One such approximation can be found in Australia’s Indigenous Cultural and Intellectual Property (ICIP) framework, which functions as an ethical protocol rather than a binding legal regime, establishing consent-based principles that require researchers and institutions to consult with relevant communities and obtain permission before using indigenous knowledge, with access conditions autonomously set by communities, potentially including usage scope restrictions, research outcome sharing, and cultural sensitivity requirements (Janke, 2021). Although ICIP lacks the enforceability of formal intellectual property legislation and operates within the settler state’s legal architecture, its significance lies in reversing the default assumption of most intellectual property regimes: rather than external legal systems classifying and allocating rights over knowledge on behalf of its holders, communities themselves define the terms of access.

The third principle is de-commodification of benefit-sharing. The normative basis for de-commodification also flows from relational sovereignty: when the value of knowledge is understood as relational, sustaining community identity and cultural transmission, rather than as exchangeable on a market, benefit-sharing mechanisms centered on monetary compensation fundamentally misrecognize the form that the value of knowledge takes. Existing benefit-sharing mechanisms rely excessively on marketized monetary compensation, which not only reinforces the commodity character of knowledge but also ignores the non-market values of traditional knowledge. Sustainable knowledge governance should explore non-market reciprocal mechanisms including knowledge exchange, capacity building, ecological restoration, and cultural respect (Morgera & Tsioumani, 2010). Brazil’s Indigenous Traditional Medicine program (Área de Medicina Tradicional Indígena), established under the World Bank-funded Vigisus II project, supported participatory action research across indigenous communities in areas including traditional birthing systems, medicinal plants, and shamanic healing practices, with the explicit goal of validating and revitalizing traditional health knowledge rather than commercializing it (Langdon & Garnelo, 2017). Peru’s “Potato Park” project, established by six Quechua communities, is dedicated to protecting traditional potato varieties and related agricultural knowledge, with funding from ecotourism revenue and international environmental organization support rather than commercial benefits from bioprospecting (Argumedo, 2008). These alternative models offer promising, if still preliminary, evidence that traditional knowledge protection may not necessarily require reliance on commodification pathways, and that non-market mechanisms can effectively support knowledge preservation and community welfare. Whether such models can be sustained and scaled across diverse institutional and political contexts remains an important question for future research. Moreover, the international patent system could address the structural cost asymmetry identified in Sect.  4 by establishing a traditional knowledge protection fund, financed through surcharges on patent applications involving biological and genetic resources and associated traditional knowledge, to support legal defense and knowledge protection efforts by Global South countries and indigenous communities. This proposal resonates with the disclosure obligations introduced by the 2024 WIPO GRATK Treaty (WIPO, 2024) and could serve as a further institutional step toward equitable knowledge governance.

The fourth principle is institutional plurality. A single global institutional framework cannot accommodate the diversity and local specificity of traditional knowledge, requiring the establishment of multi-level, polycentric governance systems. The Philippines’ Indigenous Peoples’ Rights Act recognizes indigenous communities’ collective rights to their ancestral domains, including natural resources and related traditional knowledge, granting communities autonomy to manage these rights according to their customary law (Bertrand, 2014). This arrangement allows different communities to maintain distinct knowledge management practices without uniform standards being imposed by state law. This plurality also raises a challenge: how cultural autonomy is to be reconciled with universal human rights when community customary law conflicts with state law or international human rights standards remains an ongoing tension.

The fifth principle is knowledge democratization. This requires reforming the power structures of knowledge production and validation, including research funding systems, academic evaluation standards, education systems, and policymaking processes. Freire’s systematic critique of “banking education,” in which knowledge is treated as a deposit transferred from the knowing to the ignorant, provides a foundational educational philosophy for this principle (Freire, 2000). Freire’s insistence that liberating education consists in acts of cognition rather than transfers of information, and that knowledge emerges through dialogue between equal subjects rather than through one-directional transmission, resonates directly with the epistemic equality and intercultural translation advocated in this article. The paradigm shift from “science popularization” to “science communication” that has gained wide acceptance in the philosophy and sociology of science exemplifies this Freirean logic: a move from one-directional dissemination of “correct knowledge” by scientists to the public, toward recognition of public agency in participating in the production and evaluation of scientific knowledge. This shift corroborates the present article’s core claim in the domain of traditional knowledge governance: indigenous and local communities should be recognized not as passive providers or objects of protection, but as equal participants and autonomous decision-makers in knowledge governance. In practice, such recognition requires targeted institutional support. New Zealand’s Vision Mātauranga policy offers an instructive example. Embedded across all major government research funding mechanisms since 2007, this policy requires that publicly funded research recognize and support the innovation potential of Maori knowledge, resources and people, creating dedicated funding streams for Maori-led research that operates on its own epistemological terms rather than as a supplementary input into Western research frameworks (Ruckstuhl et al., 2019). The policy demonstrates that knowledge democratization can be operationalized through systemic research funding design, though its long-term effects on epistemic hierarchies within the academy remain a subject of ongoing evaluation.

Translating these foundational principles into practice requires coordinated policy interventions across multiple governance levels. At the international level, robust polycentric governance mechanisms are essential. The current “strong TRIPS, weak CBD” configuration must be rebalanced, potentially through establishing a specialized international tribunal for traditional knowledge disputes with jurisdiction over both intellectual property and biodiversity issues; strengthening the Nagoya Protocol’s enforcement mechanisms, linking compliance with market access to create economic incentives for Northern countries; and developing international certification systems allowing consumers to identify products respecting traditional knowledge rights. The normative foundations for such rebalancing already exist. The UN Declaration on the Rights of Indigenous Peoples (UNDRIP) Article 31 recognizes indigenous peoples’ rights to maintain, control, protect and develop their cultural heritage, traditional knowledge and traditional cultural expressions (UN, 2007). Yet a considerable gap persists between normative recognition and enforceable obligation; converting these principles into binding international law through new conventions that operationalize epistemic rights requires continued effort.

Regional cooperation offers promising pathways. The Andean Community’s Common Regime on Access to Genetic Resources (Decision No. 391) represents regional-level institutional innovation, establishing unified access standards and benefit-sharing requirements across member states (Andean Community, 1996). While implementation remains uneven, this regional approach enhances Southern countries’ negotiating power against transnational corporations. The African Union’s Model Legislation for the Protection of the Rights of Local Communities, Farmers and Breeders, and for the Regulation of Access to Biological Resources attempts to establish continent-wide protection standards, though reaching consensus among diverse African countries proves challenging (African Union, 2000). These regional initiatives demonstrate that transcending national boundaries does not necessarily mean accepting Northern-dominated global regimes; South-South cooperation can create alternative transnational governance spaces.

At the national level, legislative innovation must balance multiple objectives: protecting traditional knowledge from misappropriation, promoting knowledge transmission and development, ensuring equitable benefit distribution, and respecting community autonomy. Peru’s sui generis system offers valuable lessons: its Law 27,811 establishes three knowledge categories, namely public domain knowledge (freely accessible), collective knowledge (requiring community consent), and secret knowledge (access prohibited). This classification respects indigenous communities’ own knowledge management practices rather than imposing uniform standards. However, implementation faces challenges including limited state capacity for enforcement and communities’ varying organizational capabilities.

Community-level governance remains the most critical and most challenging dimension. Genuine epistemic justice requires empowering knowledge-holding communities as primary decision-makers rather than passive beneficiaries. This demands not only legal recognition but also material support, including providing legal and technical assistance to help communities understand and exercise their rights; supporting community institution-building for effective knowledge management; facilitating inter-community networking for experience sharing and collective action; and ensuring community participation in relevant policymaking processes. Colombia’s “Biocultural Community Protocols” initiative supports communities in developing their own protocols for external actors seeking access to their knowledge and resources (Nemogá et al., 2022). These protocols articulate communities’ values, decision-making processes, and conditions for collaboration, serving as bridges between customary and state law systems.

The digital age presents both opportunities and challenges for sustainable knowledge governance. Digital technologies enable communities to document and share knowledge in unprecedented ways, potentially strengthening cultural transmission and collective identity. India’s People’s Biodiversity Registers digitally document local biodiversity and associated knowledge with community participation, creating valuable resources for both conservation and cultural preservation (Chavan & Mathur, 2020). At the same time, digitalization raises new concerns about knowledge control and privacy. Once digitized, knowledge becomes more vulnerable to unauthorized access and use, particularly with advances in data mining and artificial intelligence. The concept of “digital sovereignty” becomes increasingly relevant: communities need not just rights to their knowledge but control over the digital infrastructures that store and process it (Kukutai, 2023).

Education systems play a pivotal role in either perpetuating or transforming epistemic hierarchies. Contemporary global education systems overwhelmingly privilege Western knowledge, with indigenous knowledge marginalized or entirely absent from curricula. Sustainable knowledge governance requires fundamental educational reform: integrating indigenous knowledge as a legitimate subject of academic study rather than folkloric curiosities; training teachers in epistemic plurality and intercultural dialogue; supporting indigenous-language education that preserves knowledge embedded in linguistic structures; and establishing indigenous-controlled educational institutions where knowledge transmission follows cultural protocols. Bolivia’s “intercultural universities” exemplify such transformation, offering degree programs combining indigenous and Western knowledge systems, with curricula developed through dialogue between indigenous elders and academic scholars (Delgado & Rist, 2022). Without such structural reform in education, the epistemic hierarchies that sustain biopiracy will continue to reproduce themselves across generations, rendering legal and institutional innovations insufficient on their own.

## Conclusion

This article has examined biopiracy and traditional knowledge governance through the lenses of epistemic justice and decolonization, arguing that sustainable knowledge governance requires fundamental transformation of global epistemic power structures. The integration of Fricker’s epistemic injustice theory, Kuokkanen’s relational sovereignty concept, and Santos’s Southern epistemology reveals how biopiracy represents not merely economic exploitation but systematic epistemic colonialism perpetuating colonial relationships in the contemporary world.

The analysis demonstrates that current international frameworks, particularly the TRIPS Agreement, embody Western epistemic hegemony through seemingly neutral legal mechanisms. Requirements for individual invention, temporal novelty, and universal utility systematically exclude traditional knowledge characterized by collective creation, intergenerational evolution, and contextual embeddedness. While the CBD and the Nagoya Protocol attempt to address these inequities through benefit-sharing mechanisms, they remain constrained within property paradigms that fundamentally misrecognize traditional knowledge’s nature and value. The structural asymmetry in protection costs further compounds this injustice, requiring those whose knowledge has been misappropriated to bear the burden of their own defense.

Global South countries’ institutional innovations, from India’s TKDL to Brazil’s Biodiversity Law to South Africa’s Indigenous Knowledge Systems Policy, represent important experiments in asserting knowledge sovereignty. Yet these initiatives face structural constraints from asymmetric global power relations, Northern market regulatory gaps, internal elite capture, and emerging digital challenges. The limited success of these efforts underscores that technical or legal fixes alone cannot address deeper epistemic injustices.

This article’s primary theoretical contribution lies in demonstrating that epistemic power operates as an independent form of power within the international political economy, rather than serving merely as an ideological apparatus for material exploitation. By defining what constitutes legitimate knowledge, who qualifies as a credible knower, and what counts as innovation, Western-centric epistemic systems exclude Global South communities from the subject position in knowledge production. Biopiracy is one manifestation of this exclusion; broader epistemic colonialism operates through global education systems, academic evaluation standards, research funding mechanisms, and policy discourses. The normative framework proposed here, grounded in principles of epistemic equality, epistemic autonomy, benefit-sharing de-commodification, institutional plurality, and knowledge democratization, offers pathways beyond existing property paradigms. The reconceptualization of the knowledge subject, from individual owner to relational network, constitutes the key epistemological shift underpinning these principles.

This article also has limitations. The analysis addresses knowledge governance primarily at macro-institutional levels, with insufficient attention to micro-level community practices and everyday resistance strategies that operate outside formal legal frameworks. Moreover, due to space constraints, this article has treated traditional knowledge as a broadly unified category, when in reality indigenous knowledge systems across Africa, Asia, Latin America, and the Pacific differ considerably in their cognitive structures, social functions, and transmission modes. Future research should pursue more culturally specific analyses and develop integrated justice frameworks that connect epistemic justice with economic, racial, gender, and environmental dimensions.

The struggle over traditional knowledge governance ultimately concerns what kinds of knowledge and ways of knowing will shape collective futures. In an era of ecological crisis, the limitations of the dominant knowledge system have become increasingly apparent. Indigenous and traditional knowledge systems, far from being relics of the past, offer insights for sustainable relationships with nature that industrialized societies have yet to develop on their own. Achieving sustainable knowledge governance is therefore both a matter of justice for marginalized communities and a practical necessity for humanity at large.

## Data Availability

No new data were created or analyzed in this study.
